# Selections and Abstracts

**Published:** 1898-07

**Authors:** 


### SELECTIONS AND ABSTRACTS.

### Long, the Discoverer of Anesthesia.

The January number of *Janus,* a Dutch journal devoted principally to medical history, contains an exceedingly interesting article upon Dr. Long's discovery of anesthesia; and although we have given much of our space to this matter in the past, we know the many friends of Dr. Long still living will be pleased to see how his connection with anesthesia is viewed abroad. In fact, no one, even in America, has been a warmer friend or abler advocate of Dr. Long's claims than the author of this paper, Dr. George Foy, an eminent surgeon of Dublin, Ireland. In the English medical press within late years he has written a number of articles of a kind with the following, and we desire in this place to thank Dr. Foy for his kind interest. Dr. Foy says:

*Long, the Discoverer of Anesthesia,* is the subject of a very interesting sketch in the August-September *Bulletin of the Johns Hopkins Hospital,* of Baltimore, Maryland, America. It is contributed by Dr. Young, one of the resident surgeons of the hospital, who had the good fortune to meet Dr. Long's daughter, Mrs. Fanny Long Taylor.

The Longs of Georgia were descended, like so many of the most successful Americans, from a north of Ireland emigrant, whilst as yet the Atlantic States were English colonies. Like all his fellow. emigrants from the north of Ireland, he brought the unspeakable gifts of industry, sobriety and independence. Of such emigrants came the McDowells, the Jacksons and the McGuires—names which have become familiar as household words south of the Potomac.

It is well to bear in mind this stock from which Long is descended, for it tells much in considering the mental traits and sterling sense of honor which characterized the man.

To understand the caution and diffidence with which Long suggested the abolition of pain by etherization to his friend, Mr. James Venable, the reader must think of his prototype, the cautious, industrious, contemplative north of Ireland peasant, who contends with a poor soil and a cold, harsh, damp climate; who, withal, despairs not, and by perseverance and untiring industry compels the earth to yield him support. This being understood, we can all recognize how natural it was for Long to test the anesthetic properties of ether on every possible occasion before making known in the hamlet circle the great discovery he had made. Dr. Young might, however, have referred to this psychological factor in the object of his biography. And it would have been well to point out that at the very time when Long was pondering on the properties of ether and questioning nature for an answer, the fate of the unfortunate, learned and accomplished physician, Dr. Elliottson, of the University College, London, had become common and known. It was a dreadful objective lesson. Elliottson, like Long, sought an anesthetic, and believed he found one until he was rudely awakened from his dreams by the clear-headed Thomas Wakely, who pulled the mask from a pair of impostors.

The blow was too great for the poor dupe, and Elliottson's after-career is one of the saddest pages in English medical biography. Elliottson, honestly seeking knowledge, saw, or thought he saw, what he so ardently desired.

Long's mind was too well-balanced to fall into such an error. Nevertheless his inherited prudence called for more and more evidence of the anesthetic properties of ether until the possibility of error was eliminated; then he came forward, and, in the spirit of a Southern gentleman, and imbued with the feeling of the Great Physician, made known freely and fully, without any thought of self, the inestimable blessing of anesthesia.

All this careful scrutiny of facts and testing of results is fixed upon by Long's detractors as evidence that he failed to see the discovery he made and its beneficial effects on humanity, until Morton and the staff of the Massachusetts Hospital proclaimed it to the world—proclaimed that Morton's patent medicine "Lethon" was on sale. And all were invited to buy.

The New England Yankee was working a mine of wealth—all suffering humanity was to pay tribute to his rapacity. This audacious attempt called forth indignant protests, and amongst the most telling of these was that of Dr. Arthur Jacob, of Dublin, in his journal, the *Medical Press*.

Morton took all the necessary steps to secure a patent, but the court refused it—Long having produced anesthesia with ether four years previously, and made it a free gift to the world.

How Dr. Long came to use ether, and the story of its earlier administrations, and the documents connected with them, are told by Dr. Young with great fullness from the papers and so forth preserved with loving care by Mrs. Fanny Long Taylor, and there is little left for any succeeding biographer to add; there are, however, some slight additions which may interest the reader. Dr. Long was a personal friend of Alexander H. Stephens, and as such was heartily with the State's rights party, and, naturally, his friends in Washington were too much preoccupied in demanding Southern rights to find sufficient leisure to unravel the claims of Morton and Jackson to the discovery of anesthesia. As might be expected, Dr. Long declined to seek any favor from the enemies of his State and people.

His own people, however, recognized his claims, and the Georgia statues in the capitol are to Crawford the statesman, and Long the discoverer of anesthesia. War came, the South was invaded, and the march of Sherman's bummers laid waste a tract of thirty miles in width throughout Georgia and the Carolinas; with their marching song of hallelujah they robbed and destroyed the homes of the defenceless women and children; nothing was sacred from these wretches; they were incapable of mercy, and knew not kindness. They made, as they were instructed to do, a desolation.

Dr. Long suffered with the rest of the people in Georgia. But it is a wonderful testimony to his skill and trustworthiness that after the war the United States government, when they made Athens, Ga., a military station, appointed Long surgeon to the military hospital.

Loved by his people, he gradually made good his losses, and, at the end of a useful life, died, leaving to his family $40,000—a sum which Dr. Young may think small in comparison to Northern fortunes, but one which can hardly be looked upon as poverty. Of Long the world outside the Southern States would probably have known little if it were not for that accomplished Virginian, Dr. Landon B. Edwards, who induced Dr. Marion Sims to publish Long's biography.

The attention of the medical profession was again directed to the subject by Dr. Luther B. Grandy, Atlanta, Ga., who corrected Wilhite's story and added some new matter; and now Dr. Young gives, with a fullness never before attempted, all the literature which goes to support the claims of Dr. Crawford Long; and his paper cannot fail to interest all in the great discovery of anesthesia.—*Va. Med. Semi-Monthly.*

[One error in the above should perhaps be corrected. There are no "statues in the capitol to Crawford the statesman, and Long the discoverer of anesthesia." Under the law which permits each State to place statues of two of her distinguished sons in the National Gallery of Statues in the capitol at Washington, Georgia, twenty years ago, at the suggestion of Alexander Stephens, decided thus to honor James E. Oglethorpe and Crawford W. Long. It was found, however, that there was no provision for the expenditure of the public funds for such a purpose. So the space allowed to Georgia in the National Capitol is still vacant.—L. B. G.]

---

### Chronic Gastritis.

A report of a very severe case of gastritis was freely copied in medical journals during the year 1896, in which glycozone was successfully used.

At that time, J. W., aged 38, a blacksmith, came under my care. His illness began in 1894 with the usual symptoms of gastritis. In January, 1895, he had become so much worse that he placed himself in the hands of one of our best physicians, under whose care he continued until November of the same year, when I was consulted.

After hearing his history and the treatment given, I urged him to return to his physician, insisting that nothing more could be done. My protest was in vain.

Examination revealed an emaciated, thin and badly nourished body; his eye, skin and color fair, though pale; his temperature normal; the bowels inclined to constipation with occasional diarrhea, with white, pasty offensive stools; the lungs, heart and kidneys healthy; the liver a trifle small.

There was no painful point and no evidence of enlargement, tumor or ulcer. He was so thin that the abdomen could be most thoroughly examined. His tongue was heavily furred, red at the tip, indented at the edges, and the papillæ red and prominent.

He complained of being unable to take either solid or liquid food even in small quantities without causing heaviness, weight, oppression, pyrosis, eructation of gases, nausea and finally headache and vomiting.

Since 1894 these symptoms had increased in severity, the nausea never ceased and this whole array of complaints would gradually accumulate in force and energy, overwhelming his system with an attack of headache and intermittent vomiting that would last from three to five days.

In 1895, these storms growing worse, rendered his life almost unbearable. I had been attending him about a week, when one of these attacks occurred. He had been vomiting one day before I saw him. The scene was truly pitiable. I found my poor emaciated patient in a small darkened room scarcely able to raise his head, gagging and straining constantly, bringing up finally by the greatest of efforts, a teaspoonful of white glairy mucus; his head bound tightly or wrapped in ice cloths; his eyes congested; his cheeks hollow; his skin sallow and pale; his face bespeaking the intense agony he suffered, begging and pleading to those around him for relief from the horrible nausea and retching.

I remained with him an hour and during that time he was not free for five minutes from efforts at vomiting. His sleepless, aching brain seemed racked to distraction. He would gag, vomit, and fall back exhausted.

This continued three days, gradually lessening. Sleep came only through exhaustion. Every particle of food (liquid or solid)

was promptly vomited. During these attacks, the temperature was increased from 99 to 103.

These attacks were always of a similar character and from November 1, 1895 to July 3, 1896, they occurred every ten days or two weeks.

The physician who had treated him had used drugs, diets, and lavage faithfully and persistently, so that at the outset I was completely handicapped.

I began with the remedies which had given relief in similar cases, and in turn used acids, alkalies, alteratives, pepsin, digestants, purgatives, tonics, bitters, sedatives, diets, etc., either singly or in combination, until I had exhausted all the resources at my command.

The only perceptible relief came from the use of small doses of diluted hydrochloric acid between the attacks and a solution of cocain and morphine during the paroxysm.

About July 3, 1896, I read the article referred to above, and in desperation and despair of ever relieving him, I ordered glycozone one-half, then one drachm, well diluted, twenty minutes before meal time.

In a few days he said he felt better; within a week he repeated the assertion. To the utter astonishment of myself and his friends, one, two, four and even six weeks passed, without a recurrence of his severe symptoms.

About August 20th he was so much improved, that to hurry matters, I concluded to try lavage again. This was done at 5 P. M. and at 10 that night he was in the throes of an attack, which lasted two days.

He then resumed his glycozone and continued to improve till October 15th, when on account of inactivity of the bowels and costiveness, he was given two grains of calomel, which brought on a slight headache and considerable nausea.

He had already been taking more food, but from this time it was increased in quantity and character, eating three fairly good meals a day and enjoying them.

After beginning the use of glycozone, the acid was continued a few weeks, after meals, then left off entirely. No other medicine was used, except occasionally a pill of aloin, belladonna, strychnia, cascara, when bowels were sluggish.

To him glycozone proved the greatest boon, and to me, the relief given was simply wonderful.

It is useless to add, that I have used the remedy in many cases since, and have met with excellent and even astonishing results.—*Louis A. Kengla, M.D., San Francisco, Cal., in New England Medical Monthly, Feb., 1898.*

---

### DIAGNOSIS OF TUMORS OF THE BREAST.

In order to properly examine a neoplasm of the breast, the patient should be palpated lying down, and both mammary glands are to be carefully compared by inspection.

Now if one of the breasts is the seat of a tumor, we have to first eliminate the premammary tumors, such as a superficial abscess, lipomata and various types of erosions; and secondly, the retromammary growths, such as sub-mammary lipoma. Thirdly, the question of exostosis of the ribs, which causes the breast to project, should be considered.

We should then determine if the neoplasm is solid or liquid, and consquently its tension and fluctuation should be ascertained. If fluctuation be present, the periphery of the fluctuating pocket should be explored in order to discover if by chance it has not developed secondarily in a solid neoplasm, in which case an exploratory puncture is advisable.

If the liquid tumor is single, we would first consider the diagnosis of *galactocele*. This neoformation commences during lactation or at the time of weaning the child. It is seated in the external segment of the gland, its development is slow and gradual, it is indolent, rounded in shape and movable under the skin and over the underlying tissues. There are no enlarged glands in the axilla, while the consistency of the growth varies.

Next, we have to consider chronic tubercular abscess, the diagnosis of which is not always easy, especially at the commencement of the affection. After a time the lymphatic glands in the axilla become involved, and finally fistulæ and characteristic ulcerations will appear.

Thirdly, we have the so-called simple cysts which are due to a chronic mastitis or to epithelial neoplasms.

When we are dealing with multiple liquid tumors we are in presence of Reclus's cystic disease, which is a syndrome of either a chronic mastitis or an epithelioma. It is characterized by a number of very small tumors, which give to the feel the sensation of small lead bullets. This disease is usually bilateral, and deep or cutaneous adhesions do not exist. The lymphatic glands are not involved.

In the case of solid neoplasms we have carcinoma, sarcoma and adenoma, although pathologically the two latter growths are to be included in the same class. A very good division is *non-encapsulated tumors*, which comprises the carcinomata and *encapsulated neoplasms*, which have distinct limits, and includes the sarcomata and adenomata.

A positive diagnosis of the non-encapsulated class can be made by the age and antecedents of the patient and by a careful palpation of the tumors. In the beginning a small, hard and indolent lump, usually intimately united to the glandular structure with diffused limits and which can remain circumscribed or invade the entire gland. There soon will be found an adhesion between the growth and the skin, and in order to find this condition it is only necessary to pick up a fold of the skin overlying the neoplasm between the thumb and index-finger when a puckering of the skin is to be seen and felt. In fact the aspect is that of orange peel.

To ascertain if the growth is adherent to the aponeurosis of the pectoralis major, the former should be seized with the hand and the muscle made to contract.

Other later signs of the disease are retraction of the nipple, a discharge of serum or blood from the nipple. When blood comes from the nipple the neoplasm is in all probability an intra-canalicular epithelioma. As development of the growth increases, the axillary glands become involved and sometimes the sub-clavicular or the axillary glands of the opposite side can present an infiltration.

When well advanced the neoplasm first presents a small ulcerating surface, which is quite superficial, but which invades in depth little by little and gives rise to considerable hemorrhage. The borders of the ulceration become hard but not undermined; the bottom of the ulcer is uneven and secretes a fetid, sanguineous discharge. Pain is also complained of by the patient and is caused by the compression of the nerve trunks in the axilla or by direct neoplastic infiltration of the nerves, and to end the clinical picture, cachexia makes its appearance.

As to the differential diagnosis, we have to consider the two types of chronic mastitis—viz., mastitis with multiple nodules, which are more likely to be mistaken for an adeno-fibroid than carcinoma, and a mastitis presenting only a single nodule. Diagnosis is made by the patient's antecedents (lactation), the oscillations in the size of the growth, the earlier and greater involvement of the axillary glands, the pain and the frequent involvement of both breasts.

Mammary tuberculosis is an infrequent disease, and the diagnosis is not difficult if the subject presents other symptoms of the disease, but when localized in the breast it might cause great difficulty in the diagnosis.

Tertiary syphilis of the breast is infrequent and can only be diagnosticated by a history of syphilis, or in want of this by specific treatment.

The shape and limits of the mammary tumor are to be considered carefully. We have circumscribed tumors; the ordinary so-called scirrhus; a form of scirrhus sending out ramifications in all directions, which start from the center of the growth and are adherent to the skin; the atrophying scirrhus has a slow evolution and causes the gland to shrivel up, while the lymphatic glands are involved at a late period, and ulceration of the neoplasm is tardy and superficial.

Encephaloid differs from ordinary scirrhus in that it is of a lesser consistence and cysts may develop within the tumor, but its progress is far more rapid.

Disseminated pustular scirrhus is characterized by the presence of multiple cancerous growths in the skin and subcutaneous cellular tissue; its prognosis is very serious. Scirrhus *en cuirasse* is the condition met with when the entire thorax is invaded by large patches of the growth. It is hard and violet in color, producing much difficulty in respiration, and is rapid in its progress.

Volkmann's carcinomatous mastitis occurs in young women during lactation and from the beginning invades the entire gland, in many instances both; it progresses with great rapidity and cachexia appears early, while death results in a few months.

The histological diagnosis is often of considerable difficulty, but we have certain clinical characters which will aid us in differentiating a canalicular epithelioma from a mastitis.

The encapsulated neoplasms are to be recognized by their mobility over the gland, by the absence of adhesions with the skin, and underlying tissues and the glands in the axilla are not involved.

Adenoma is a rather small tumor, round, smooth, very regular in outline, indolent; its consistency is elastic. Generally, several of this neoplasm will be found in one gland, and the diagnosis is only to be mistaken for multiple nuclei or a chronic mastitis. The latter affection can be diagnosed by the time of occurrence, the pain and the early enlarged and painful glands in the axilla. The histological diagnosis of the type of adenoma present in a given case can only be made after removal of the growth.

A sarcoma is a smooth, indolent tumor which is small in size in the beginning. But suddenly it takes on a rapid growth, and when developod it must be diagnosticated from carcinoma. The diagnosis is based upon the progress and manner of development, which is slow at the commencement, and then suddenly rapid, as well as on the exterior shape of the growth, which is lumpy and projecting; on the late adhesion to the skin, which takes place *en masse;* by the presence of large subcutaneous veins. *In carcinoma we sometimes meet with a varicose condition of the lymphatics, but the veins are not dilated.* In sarcoma, the skin is shiny and distended and the nipple is spread out and not retracted as it is in most cases of carcinoma. The characters of the ulceration are next to be considered.

Carcinoma is uniformly hard in consistency, while sarcoma is semi-solid or semi-liquid, and enlarged glands in axilla are not present in this latter neoplasm. Sarcoma has a tardy influence on the patient's general health and is itself usually indolent.

Recurrence after removal is common to both carcinoma and sarcoma, but differs in that the neoplasm returns at the site of the former growth in sarcoma, while in carcinoma the affection appears in the lymphatics. In sarcoma the seconda ry foci occur in the lungs and liver more especially, the disease being disseminated by the general circulation.

Surgical interference is indicated in the majority of mammary tumors by free extirpation of the growth and dissection of the masses of glands in the axilla. Adenoma and sarcoma require only a free extirpation. Benign tumors of the breast should be removed, as they may undergo a malignant degeneration at a later period.—*Annals of Gynecology and Pediatry.*

---

### APHTHOUS STOMATITIS.

A. Levi (*Wiener Medic. Blätter, 1897, xx, 39*). Stomatitis aphthous, according to most authors, is an acute inflammation of the mucous membrane of the mouth, characterized by a fibrinous deposit in the form of white or yellow spots of the size of a hempseed to that of a lentil. Billard and others consider this disease an inflammation of the mucoid follicles of the mouth. Vogel and Biedert look upon aphthæ as ulcerations, and divide them into aphthous and ulcerative stomatitis. Rilliet and Barthez consider them to be vesicles, which later on became ulcers (Stomatite vésicoulcéreuse of Rilliet and Barthez). Monti describes aphthous stomatitis as an inflammation of the mucous membrane of the mouth, which causes fibrinous deposits on and under the epithelium. In the author's opinion this explanation, as we shall learn from pathologico-anatomical investigations, is the only correct one.

*Etiology and Pathogenesis.*—Aphthous stomatitis is a disease of childhood. In the adult it is rarely met with, and then usually in women during the menstrual period, in child-bed and during lactation. From the statistics of 111,143 cases we may form the following conclusions :

1. That aphthous stomatitis is present in children in 0.972 per cent.—*i. e.*, about 1 per cent.

2. That the disease more frequently attacks females than males· Levi does not believe that this circumstances, which has already been remarked by Monti and others, should be given any particular importance.

The disease is found in children of every constitution. He is not able to agree, from his experience of ninety cases, with Billard, Rilliet and Barthez, in their opinion that weakly and lymphatic children are peculiarly predisposed. Monti indeed has observed, and Levi confirmed it, that this disease is frequently met with in rachitic children. He does not believe, however, that this is due to a weak constitution, but is due to disorders of digestion to which these children are subject, and, as Monti suggests, to the great frequency of carious teeth. As regards the age, aphthous stomatitis is found even in infants at the breast; nevertheless it is very rarely met with before the period of dentition. Monti has never met with it in the new-born. In 496 cases which Levi has gathered he found the disease once in a child fourteen days old, and once in a child twenty-eight days old. Bednar found one case in 100 new-born infants; Bohn, three in 169 during the first month after birth; other cases are mentioned by Denis and Billard. Levi agrees with Monti that these cases were probably not true aphthous stomatitis, but cases of simple isolated aphthæ. Its rare occurrence before the period of dentition is probably due to the uniform nutrition during the period of infantile life, and to the greater care observed by the mothers in regard to the cleanliness of the infant's mouth.

The inflammation of the mucous membrane, the increase in the secretion of saliva, the raised temperature of the mouth—which frequently accompany detention—are conditions favorable to the development of micro-organisms and to the decomposition of food.

The season of the year also seems to play an important rôle in the etiology of aphthous stomatitis, for in the warmer months this disease is met with most frequently. Another important factor is the relation of this disease with epizootic aphthæ. Some authors identify both diseases and assign infection through the milk of animals suffering from aphtha epizootica as the only cause of the disease.

Levi was fortunate enough to be able to demonstrate that this stomatitis occurred in children in whom an infection through milk could be positively excluded, as the latter was always carefully sterilized before use.

Aphthous stomatitis is a contagious affection; drinking glasses, utensils, soiled clothing, etc., may become carriers of infection.

*Diagnosis.*—Bearing in mind that vesicles only are found and that the process of disease consists in depositing a fibrinous exudate in the shape of macules on different parts of the mucous membrane, it will be easy to make a diagnosis. Mistaking it for ulcerative stomatitis may, however, occasionally occur, as these two processes frequently exist together. The aphthæ, however, consist of an inflammation which remains localized on the superficial portion of the mucous membrane of the mouth with the deposition of a fibrinous exudate, while in ulcerative stomatitis, necrosis and loss of substance is characteristic of the disease, because we have to deal in the latter with a diffuse parenchymatous inflammation followed by an ulcerative necrosis. Aphthous stomatitis is exceedingly painful; ulcerating stomatitis gives the patient only a sensation of swelling and pulsation. From sprue, it may be distinguished by the course of the disease and by a microscopical examination. In Bechar's aphthæ, the seat of the aphthæ and the age of the patient will lead us to avoid mistakes.

*Prognosis* is favorable in the greater number of cases. It may, however, become very, very grave when an edema of the glottis is occasioned by the formation of an excessive number of aphthæ in the posterior portion of the throat and in the confluent epidemic form.

*Treatment.*—Aphthous stomatitis being an infectious disease, isolation of the patient is of the greatest importance in its prophylactic treatment. The milk should always be boiled or sterilized. The general condition of the child should be carefully looked after. Local or general disease (for example, bronchitis, gastroenteritis, eczema, etc.) should at once be combated, for the greater number of cases of stomatitis is observed in these cases. The hygiene of the mouth is of the highest importance, as without doubt various micro-organisms will be formed here from the decomposition of food particles, etc. In the second year of life, and during the period of dentition particularly, the hygiene of the mouth should be observed with the greatest care, as the children at this time are not yet able to masticate thoroughly, and swallowing of all food particles are not perfectly accomplished, so that remnants of food frequently remain in the mouth. In fever patients also disintegration of the food occurs more rapidly. In these cases Vichy water or the following has a very good effect:

| | | |
|---|---|---|
| ℞ | Sodii bicarbon | 3.0 |
| | Sodii borac | 2.0 |
| | Aquæ destill | 100.0 |

Mix.

Antimycotic remedies, as salicylic acid, benzoic acid, boric acid, etc., are also to be recommended. Treatment should be both local and general. Locally the following solutions are recommended, with which the diseased portion may be painted four or five times a day:

| | | |
|---|---|---|
| ℞ | Sodii borax | 4.0 |
| | Tinct. myrrhæ | 8.0 |
| | Syr. morerum | 60.0 |

or,

| | | |
|---|---|---|
| ℞ | Borac | 4.0 |
| | Tinct. benzoe | 2.0 |
| | Aq. destill | 10.0 |
| | Syr. simpl | 20.0 |

or,

| | | |
|---|---|---|
| ℞ | Sodii phosph | 10.0 |
| | Aq. naphæ | 25.0 |
| | Mellis rosati | 50.0 |

or,

| | | |
|---|---|---|
| ℞ | Calc. chlor | 2.0 |
| | Mellis | 20.0 |

or,

| | | |
|---|---|---|
| ℞ | Potas. chlor | 3.0 |
| | Aq. destill | 60.0 |

Baginsky recommends potassium permanganate (0.1 to 15.0), and claims a specific action for it. Hirtz recommends

| | | |
|---|---|---|
| ℞ | Acid. salicyl. | 2.0 |
| | Alcohol | 10.0 |
| | Glycerin | 20.0 |

or,

| | | |
|---|---|---|
| ℞ | Sodii salicyl | 20.0 |
| | Aq. destill | 100 |

Most cases will yield to any of these formulæ or heal even without treatment. Again, we meet with those which, in spite of early treatment, present a grave form of the disease as early as the second day.

Levi, from the observation of a great number of cases of stomatitis, maintains that no other remedy will give the same favorable results that chloric potash attains. It may be looked upon as a specific. Its external use should be combined with its internal administration to obtain the very best results.

Monti's prescription is:

| | | |
|---|---|---|
| ℞ | Potas. chlor. | 4.0 |
| | Aq. destill. | 200.0 |
| | Tinct. myrrhæ | 3.0 |

M. Sig.—For washing out the mouth.

and

| | | |
|---|---|---|
| ℞ | Potas. chlor. | 1.0 |
| | Aq. destill. | 90.0 |
| | Syr. rubi idæi | 10.0 |

M. Sig.—Keep on ice. A teaspoonful every two hours.

In stubborn and severely painful cases it will be necessary to use a solution of salicylic acid, according to Hirtz, or of corrosive sublimate.

| | | |
|---|---|---|
| ℞ | Hydrarg. bichlor. corr. | 0.1 |
| | Aq. destill. | 50.0 |

M. Sig.—For penciling the mouth.

Should this, however, fail, or should the aphthæ pass into an ulcerative form, a superficial cauterization with lapis mitigatus or sulphate of copper must be resorted to. Where pain is exceedingly great, solutions of the tincture of opium, of extract of opium or of cocain may be applied. Only cold liquid nourishment should be given, and this, especially in cases of recurrent aphthæ, which are nearly always accompanied by a state of irritation of the gastro-intestinal mucous membrane, should be as nutritious as possible.—*Pediatrics.*

### FAMILY HISTORY IN RELATION TO LIFE INSURANCE.

The *age at death* of parents and grandparents is important as indicating the strength or weakness, endurance or feebleness of constitution of the applicant, and his probable longevity. It is an accepted maxim that long life runs in families, and that an inherited tendency to attain old age is the primal qualification for longevity. In other cases the opposite tendency is shown, and often every individual of the family dies before reaching middle life. It is no exaggeration to say that even a weak and puny child of a long-lived race is more apt to reach three score and ten than is a strong and vigorous child of a short-lived race.

A rough and ready method of computing an applicant's probable duration of life consists in taking the sum of his parents' and grandparents' ages at death, and dividing by six. The quotient indicates the age to which the applicant may reasonably expect to live; the presumption being that a man who has reached maturity in good health ought, with proper care, to attain the average age of his parents and grandparents. This method serves as a measure of his constitutional vigor, the amount of capital he has in the bank of life. For example, I remember once examining an applicant, the sum of whose parents' and grandparents' ages, divided by six, was eighty-three years; while the man himself was one of a family of eleven children, every one of whom was then living and in good health. Such a man would be rated as a desirable risk by any insurance company, and there would be a tendency on the part of the medical officers to overlook any minor disabilities or unfavorable circumstances which might cause the rejection of an applicant of less favorable heredity.

In case either of the parents or grandparents should be living and in good health, and also where it can be clearly shown that the death occurred from purely accidental causes, not in any way indicating weakness of constitution, it may be safe to correct the result, adding to the age of the living or accidentally dead, the expectation of life for the given age, according to the table.

The age of brothers and sisters is also important, and furthermore the age of the applicant's children, if any—though this last is seldom or never called for by insurance blanks—as indicating whether the vitality of the earlier generations is being preserved, or increased, or diminished. For example, if the average age of the grandparents was eighty, and of the parents sixty, while most of the applicant's brothers and sisters died young, and his children were feeble or died in infancy, the outlook for long life on his part would be adjudged far less favorable than would be the case if opposite conditions prevailed, and the average longevity of the successive generations was increasing.

The *cause of death* has probably had more importance attached to it than is justly its due. Especially is this the case when the age is not young. In any case, the important point with the insurance companies is not what particular disease the insured may die of, but simply that they may live out their expectation, and if possible, go on beyond that to a green old age, not how long he may be sick even, but how long he will live. If an insured person reaches seventy years of age, it matters little whether he dies of consumption, or pneumonia, or accident. If, however, he dies before forty, his death is presumably due to a lack of endurance, vigor, and vitality. One may be ill and suffer many years, and yet have a strong constitution. Indeed it is the strong constitution which enables its possessor to live and endure suffering, while a weaker person would die at the first severe attack of disease.

It has usually been assumed that there is an especial tendency on the part of the child to die from the same disease which caused his parent's death, in cases where this disease was of the class known as hereditary. Modern researches have proved conclusively, however, that it is not so much diseases as tendencies that are inherited, and that the tendency is not always to a particular disease, but may consist in a weakness of certain organs or systems, which constitute an increased liability to certain great classes of disease. Especially they have shown, in the case of consumption, that the child of consumptive parents does not so much inherit tuberculosis, or even an especial tendency thereto, as he does a weakened constitution, a lack of resisting power, which makes him an easy prey to any form of wasting disease—the special form which it will take in his case, or whether, indeed, he may escape entirely, to be determined by his particular surroundings and habits of life—in short, by his environment. Furthermore, the present tendency is to look upon many of the cases of consumption which were formerly regarded as hereditary, as the result of contagion. There is a wide field for research in this direction.

Again, the liability to alcoholic or narcotic inebriety may be the result, not merely of the like inebriety in the parent or grandparent, but of epilepsy, hysteria, neurasthenia, melancholia, insanity or any form of nervous instability. There is, indeed, a system of reciprocity between the principal forms of hereditary disease, whereby each may result from the other, under certain conditions. The great essential in family history from an insurance point of view, is that the applicant should have inherited a good degree of constitutional vigor. Environment tells the rest.—*Dr. J. M. French, in Medical Examiner.*

---

### YELLOW FEVER AND THE WAR.

The report published last week that yellow fever had developed in members of a prize crew on one of the captured Spanish vessels held at Key West naturally causes some apprehension at this particular time. "Yellow Jack," always a most unwelcome guest, would be especially so now, despite the excellent facilities at the command of the Marine Hospital Service for preventing or stamping out an epidemic. The great importance, however, to the United States of the yellow fever question lies in the fact that there will probably be an invasion by our troops of territory in which the fever prevails each year with considerable virulence. It will be well for us, therefore, to learn our lesson from past rather than from present and future experience. We must not forget that armies have been decimated in the West Indies without a man being lost at the hands of a human enemy. A century ago, according to John Hunter, who was a surgeon in the English army in Jamaica, scarcely a man was left in some regiments at the end of a year, although the number killed in battle was almost nothing.

All authorities agree upon this point: that it is the newcomer, and especially the unacclimated soldier, who suffers to the greatest extent. The ravages of former times among European troops are hardly to be anticipated in an American army of invasion, because we now know that hygienic conditions which military authorities can control have much to do with keeping down the death-rate. Our soldiers would, for example, not be landed in an impaired state of health after long voyages in overcrowded vessels. The food supply would be much superior to that of former days, and quarters would be so far as possible chosen at a distance from swamps, while the water supply would be much more carefully controlled. Overcrowding and lack of sufficient air space have been important causes of the high death-rate in barrack and hospital in former West Indian campaigns—conditions not now so likely to exist, since their danger is so well appreciated.

In spite, however, of all that is known of the dangers of yellow fever and of the means of prophylaxis, many deaths might occur among our men from a lack of knowledge of proper personal conduct under the new conditions of life. It would be well, then, that each man should receive instruction as to his mode of living. In a little book, just published in London, on "Yellow Fever in the West Indies," the author, Dr. Izett Anderson, states that after an experience of more than thirty years he can say that undue exposure to the sun in the heat of the day is the most potent exciting cause, and next to this are intemperance and debauchery.

Earth freshly upturned, as in the digging of trenches, is a source of danger which it might at times be impossible to guard against. Past experience, however, should guide in sending troops into a yellow fever region to engage in such work, since it has been found that the most susceptible subjects come from the more northerly and colder regions, while colored-skinned races are but slightly endangered. Then, too, those who have acquired immunity by previous exposure should be first utilized.

We cannot here enter into the importance of an early diagnosis of the first case in a community, or of the difficulties at times attending the detection of the affection by physicians who have had no practical experience with the fever. The author above referred to dwells especially upon the dangers of faulty diagnosis—in instances in which the disease is seen for the first time by the physician—in the stage of depression or decline of the fever, when the symptoms may be limited almost to cutaneous capillary hyperemia and conjunctival injection, and when the patient expresses himself as feeling well again. The treatment he recommends embraces an attempt at abortion of the process by administering twenty grains of calomel, together with twenty or more grains of quinine, and repeating the dose if necessary at an interval of three or four hours; and if the fever remains high, even a third dose may be given. This must be employed early in the first stage, and is particularly suited to sthenic cases in plethoric soldiers, especially those who have recently arrived in the infected region.

As a routine treatment he has employed for many years:

| | |
|---|---|
| Acidi carbolici | gr. xviij. |
| Potassii bicarbonatis | ʒ iij. |
| Aquæ | ℥ xij. |

Of this two ounces are to be given well iced every two or three hours, a dessertspoonful of freshly squeezed lime juice being added to the effervescing mixture at the time of taking.

Among other measures recommended are wet packs to the body; "iced drip" to the head; dry cupping to prevent or relieve suppression, together with drachm doses of spirits of nitrous ether every hour; and bromide of ammonium in full dose given in iced milk for insomnia. He speaks favorably, too, of Sternberg's formula:

| | |
|---|---|
| Hydrargyri bichloridi | gr. $\frac{1}{3}$ |
| Sodii bicarbonatis | gr. cl. |
| Aquæ | ℥ xl. |

S. Three tablespoonfuls given every hour, given ice-cold.

It is gratifying to note that the government is taking most active measures to solve this great war problem, and that an officer of the Marine Hospital Service has been detailed to accompany the army of invasion, whose special duty it shall be to secure for our soldiers all the protection possible from this hidden enemy. Among the measures to be carried out are the establishment of a base of supplies at a distance from any yellow fever center, and prevention of communication between the army and infected localities.

The latter will naturally be a most difficult thing to accomplish. The only absolute safety would seem to lie in terminating the war in Cuba before June, when the annually recurring epidemic in and about Havana makes its appearance, or in so far accomplishing this end that the native army, assisted by immunes, could complete the work and permit susceptible northern troops to return to the States.

We have the utmost confidence in the ability of our Marine Hospital Service to accomplish, by vigilance and scientific sanitation, all that it is possible to do in the matter of detecting early evidences of danger and carrying out segregation. A disease, however, which presents such an appalling mortality in severe epidemics and under certain condtions should likewise be regarded as an enemy to be combated until conquered. No doubt this will be one of the results of the present warfare, for it may be predicted that with Havana under United States control the unwholesome conditions which have permitted this yearly visitation of yellow fever will be done away with and the city made as safe from June to November as it is from November to June.

It has been pointed out by authorities, and former experience in our own country has shown it to be a fact, that old wharves with their rotting timbers, unpaved streets, collections of decaying vegetable matter, and lack of proper sewerage, all contribute to epidemic outbreak. Such sanitary defects are abundant in Cuba's capital, and it will become our duty at no distant day to fortify the city by modern hygienic improvements for the benefit of humanity at large.—*Gaillard's Med. Journal.*

---

### PROCEEDINGS OF THE ST. LOUIS MEDICAL SOCIETY.

*Meeting of Saturday Evening, February 5, 1898.—President Dr. J. C. Mulhall in the Chair.*

*Dr. Keating Bauduy* read a paper entitled "Observations on the Treatment of Some Cases of Neurasthenia," written by Jerome K. Bauduy. The doctor also read a paper giving "Microscopical Report," by Dr. C. Fisch. Also "Clinical Report," by himself. (See *Review* of February 26, 1898, page 146.)

### DISCUSSION.

*Dr. Stoffel.*—I would like to ask Dr. Bauduy whether he does not think the dieting of patients and placing them in hygienic surroundings had not as much to do with the results as his medicine.

*Dr. Fairbrother.*—I would like to ask another question: If this "Pepto-Mangan" is not in the class of proprietary medicines?

*Dr. Keating Bauduy.*—I will endeavor to respond to the questions propounded. Dr. Stoffel wants to know if the dietetic and hygienic measures alone being adopted would not have effected a cure in the cases reported. I will state that in many of these cases we have tried other preparations of iron and with rather negative results; and in all these cases we have observed hygienic and dietetic indications without obtaining these remarkable improvements. Now I do not wish to be understood that this remedy is a panacea; I merely give you the data and clinical facts, and the results of the microscopic investigation, and you can take them for what you believe them to be worth.

I will answer Dr. Fairbrother by saying that I presume that this is a proprietary remedy, but I use a good many other proprietary preparations. I use antipyrine, and I suppose the doctor does; I use phenacetine, sulfonal, and other such proprietary remedies, and I will tell you candidly, gentlemen, that I use whatever I find benefits my patients. Of course I do not propose to use nostrums or remedies of which we know nothing about their composition. But the Gude preparation of iron does not belong to this class; a great many gentlemen here use it; I use it because it is the best remedy that I have obtained for the treatment of these cases.

*Dr. Johnston.*—There is no iron in it, is there, doctor?

*Dr. Keating Bauduy.*—Yes, sir, there is iron in it; in the form of a peptonate of iron.

*Dr. Jerome K. Bauduy.*—One salient feature of this paper which has not been brought out as prominently as it might have been, on which I wish to lay particular emphasis, is that whether it be a proprietary remedy or not, matters not provided it cures our patients. It is our business to cure our patients, it matters not by what means. But the point is this, that it is my opinion, based upon observation in these cases, that we have not paid sufficient attention to the *organic* salts of iron; in other words, that the other preparations of iron do not produce the results that these organic preparations achieve. For years the combination of iron and manganese I have used in daily practice. I have used a great many of these preparations and the great point has been to obtain one which is assimilable, that is elegant, and that will not produce anorexia and other gastric disturbances. Now with the organic salts of iron we have had startling results, and I intend to use them as long as they benefit my patients. I do not wish to be understood by the neurologists and others present as saying that this is a proper remedy for all cases of neurasthenia, but I do maintain that it is a remedy well suited to those neurasthenic and anemic cases described, especially in women suffering with menstrual irregularities, especially those accompanied by hemorrhage. I simply want the gentlemen to judge by the results. "Facts speak louder than words." "*Facta non verba.*"—*Medical Review*, March 12, 1898.

